# Cannabinoid Receptor 1 Agonist ACEA and Cannabinoid Receptor 2 Agonist GW833972A Attenuates Cell-Mediated Immunity by Different Biological Mechanisms

**DOI:** 10.3390/cells12060848

**Published:** 2023-03-09

**Authors:** Nuchjira Takheaw, Kanyaruck Jindaphun, Supansa Pata, Witida Laopajon, Watchara Kasinrerk

**Affiliations:** 1 Division of Clinical Immunology, Department of Medical Technology, Faculty of Associated Medical Sciences, Chiang Mai University, Chiang Mai 50200, Thailand; 2 Biomedical Technology Research Center, National Center for Genetic Engineering and Biotechnology, National Science and Technology Development Agency, Faculty of Associated Medical Sciences, Chiang Mai University, Chiang Mai 50200, Thailand

**Keywords:** endocannabinoid system, cannabinoid receptors, cannabinoid receptor agonists, T cell responses, immunoregulation

## Abstract

Cannabinoid receptor 1 (CB1) and cannabinoid receptor 2 (CB2) are components in the endocannabinoid system that play significant roles in regulating immune responses. There are many agonists for the cannabinoid receptors; however, their effects on T cell regulation have not been elucidated. In the present study, we determined the effects of the CB1 selective agonist ACEA and the CB2 selective agonist GW833972A on T cell responses. It was found that both agonists impaired anti-CD3 monoclonal antibody induced T cell proliferation. However, ACEA and GW833972A agonists down-regulated the expression of activation markers on CD4^+^ and CD8^+^ T cells and co-stimulatory molecules on B cells and monocytes in different manners. Moreover, only GW833972A suppressed the cytotoxic activities of CD8^+^ T cells without interfering in the cytotoxic activities of CD4^+^ T cells and NK cells. In addition, the CB2 agonist, but not CB1 agonist, caused the reduction of Th1 cytokine production. Our results demonstrated that the CB1 agonist ACEA and CB2 agonist GW833972A attenuated cell-mediated immunity in different mechanisms. These agonists may be able to be used as therapeutic agents for inducing T cell hypofunction in inflammatory and autoimmune diseases.

## 1. Introduction

The endocannabinoid system is composed of endocannabinoids, cannabinoid receptors, and the enzymes responsible for the synthesis and degradation of the endocannabinoids [1,2]. Two primary endocannabinoid receptors, cannabinoid receptor 1 (CB1) and cannabinoid receptor 2 (CB2), have been identified. Both receptors constitute a class of cell membrane receptors in the G protein-coupled receptor family [1,2]. Endogenous ligands for the cannabinoid receptors are biologically active fatty acids, including N-arachidonoylethanolamine (anandamide (AEA)) and 2-arachidonoyl glycerol (2AG) [3,4]. Although the endocannabinoid system is broadly expressed in almost all organs throughout the body, the expression levels of cannabinoid receptors are highest in the nervous and immune systems [5]. The endocannabinoid system has been demonstrated to play important roles in various biological and pathological conditions, including the regulation of cognitive and immune responses [6,7,8]. In the immune system, immune cells highly express cannabinoid receptors and also produce endocannabinoids. The effect of endocannabinoids in innate and adaptive immune responses have been demonstrated [8,9,10,11,12]. Upon reacting to their ligands, CB1 and CB2 receptors are activated and cell signaling occurs, resulting in the regulation of T cell functions [10,11,13,14,15]. However, CB1 and CB2 activation by various types of agonists showed different outcomes on the regulation of CD4^+^ and CD8^+^ T cell responses.

The cannabis plant contains numerous compounds, including cannabinoids [16]. These phyto-cannabinoids were demonstrated to activate the cannabinoid receptors and affect various human biological activity, including the immune system [17,18]. Accordingly, phyto-cannabinoids and cannabis-based treatments have attracted attention as pharmacological agents for the treatment of several disorders. However, various adverse effects of using these substances have been observed [19,20]. This evidence precludes the widespread use of phyto-cannabinoids and cannabis in the clinic. Substantial efforts have thus been focused on developing synthetic cannabinoid receptor selective agonists, which specifically target either CB1 or CB2, for use as therapeutic agents with less adverse effects.

Currently, several CB1 and CB2 selective agonists have been developed [21,22,23]. The synthetic cannabinoid receptor agonists are different in chemical structure and function. Their structural characteristics permit them to interact with CB1 or CB2 expressed on the cell surface [24]. Some of these synthetic agonists were demonstrated to modulate the immune function and have therapeutic potential for several disorders [21,25,26]. New cannabinoid selective agonists are currently the focus of academic and commercial attempts. However, the effect of many cannabinoid receptor agonists on T cell regulation have not been elucidated. In this study, therefore, we focused on studying the effects of the CB1 selective agonist ACEA [27] and CB2 selective agonist GW833972A [28] on the regulation of CD4^+^ and CD8^+^ T cell responses. The effects of ACEA and GW833972A on T cell responses have not been reported. Hence, the present study provides the poly-pharmacological properties and therapeutic potential of ACEA and GW833972A.

## 2. Materials and Methods

### 2.1. Antibodies

Anti-CD3ε mAb (clone OKT3) (Ortho Pharmaceuticals, Raritan, NJ, USA) and anti-CD28 mAb (clone L293) (BD Bioscience, San Jose, CA, USA) were used. PE-conjugated anti-CD25, anti-PD-1, anti-LAMP-1, isotype-matched control mAbs, FITC-conjugated anti-CD4 mAb, APC-conjugated anti-CD8 mAb, and PEcy7-conjugated anti-CD19 mAb were purchased from BD Bioscience. PEcy7-conjugated anti-CD3 mAb, PE-conjugated anti-CD14 mAb, and PE-conjugated anti-IL-2, anti-IFN-γ, and anti-TNF-α mAbs (BioLegend, San Diego, CA, USA) were used. PE-conjugated anti-CD69 mAb and FITC-conjugated anti-CD86, CD80, HLA-DR and HLA-ABC mAbs (ImmunoTools, Friesoythe, Germany) were used. FITC-conjugated isotype-match control mAb were produced in our laboratory.

### 2.2. Reagents

Arachidonyl-2′-chloroethylamide hydrate (ACEA; CB1 agonist), Rimonabant hydrochloride (SR141716A; CB1 antagonist/inverse agonist), GW833972A (CB2 agonist), SR144528 (CB2 antagonist/inverse agonist), and dimethyl sulfoxide (DMSO) (Sigma-Aldrich, St. Louis, MO, USA) were used. These cannabinoid receptor agonists and antagonists were dissolved in DMSO at a concentration of 20 mM and stored at −20 °C until used.

Saponin (Amresco, Solon, OH, USA) was used. Carboxyfluorescein succinimidyl ester (CFSE), brefeldin A, and monensin were obtained from Sigma-Aldrich. RPMI 1640 medium and fetal bovine serum (FBS) (Gibco, Grand Island, NY, USA) were used. Ficoll-Hypaque solution (IsoPrep)(Robbins Scientific Corporation, Sunnyvale, CA, USA) was used. Finally, 7-AAD solution was purchased from BioLegend.

### 2.3. Cells

P815 cell line (gift from Prof. Dr. Seiji Okada, Kumamoto University, Kumamoto, Japan) and K562 cell line (acquired from ATCC) were maintained in RPMI-1640 medium supplemented with 10% heat-inactivated fetal bovine serum (FBS), 40 μg/mL gentamycin, and 2.5 μg/mL amphotericin B (10%FBS-RPMI 1640) at 37 °C in a humidified 5% CO_2_ atmosphere.

Peripheral blood mononuclear cells (PBMCs) were isolated from heparinized whole blood or buffy coat of healthy individuals obtained from The Thai Red Cross Society, Chiang Mai, Thailand, using standard Ficoll-Hypaque density gradient centrifugation.

### 2.4. Cell Proliferation Assay

CFSE dilution technique was used for cell proliferation assay. PBMCs at 1 × 10^7^ cells/mL were labeled with CFSE at 1 μM final concentration. The labeled cells were washed and re-suspended in 10% FBS-RPMI 1640.

For investigation the effect of CB1 and CB2 agonists on T cell proliferation, CFSE labeled PBMCs at 1 × 10^5^ cells (at 100 µL final volume) were stimulated with immobilized anti-CD3 mAb clone OKT3 (12.5 ng/mL) or kept unstimulated in the presence of various concentrations of ACEA (CB1 agonist), GW833972A (CB2 agonist), or relevant concentrations of DMSO at 37 °C in 5% CO_2_ incubator for 5 days. Cells were, then, harvested for measuring the CFSE reduction by a flow cytometer (BD Accuri^TM^ C6, BD Biosciences) and analyzed by FlowJo software.

For testing the effect of CB1 and CB2 antagonists on CB1 and CB2 agonists, CFSE labeled PBMCs at 1 × 10^5^ cells (at 100 µL final volume) were cultured with immobilized anti-CD3 mAb clone OKT3 (12.5 ng/mL) or medium in the presence of SR141716A (CB1 antagonist) or SR144528 (CB2 antagonist) for 15 min at room temperature. Then, ACEA, GW833972A, or relevant concentrations of DMSO were added and cultured for 5 days in 5% CO_2_ incubator. T cell proliferation was determined by a flow cytometer (BD Accuri^TM^ C6).

### 2.5. Assay for T Cell Activation Markers

PBMCs at 1 × 10^6^ cells (at 1000 µL final volume) were cultured with or without immobilized anti-CD3 mAb in the presence of CB1 or CB2 agonists or relevant concentrations of DMSO. After incubation, the cells were stained for CD69 and CD25 markers at day 1 and PD-1 marker at day 3. Briefly, the Fc receptors on the harvested cells were blocked with PBS containing 10% human serum (blood group AB). Cells were stained with PE-anti-CD69, -CD25, -PD-1 mAb, or isotype-matched control mAb. Then, cells were fixed and permeabilized with 4% paraformaldehyde and 0.1% saponin in PBS containing 5% FBS-0.1% NaN_3_. The FITC-anti-CD4 mAb, PEcy7-anti-CD3 mAb, and APC-anti-CD8 mAb were added into the cells and incubated for 30 min. The stained cells were determined by a flow cytometer (BD Accuri^TM^ C6). Gating strategy was shown in Supplementary Figure S1.

### 2.6. Assay for Co-Stimulatory Molecules on Monocytes and B Cells

PBMCs at 1 × 10^6^ cells (at 1000 µL final volume) were cultured with or without immobilized anti-CD3 mAb in the presence of CB1 agonist, CB2 agonist, or DMSO in a 5% CO_2_ incubator for 18 h. The Fc receptors on the harvested cells were blocked with PBS containing 10% human serum (blood group AB) and stained with PE-anti-CD14 mAb, PEcy7-anti-CD19 mAb, and each FITC-mAbs (anti-HLA-ABC, -HLA-DR, -CD80, or -CD86 mAb) or FITC-isotype-match control mAb. The expression levels of co-stimulatory molecules on CD14^+^ monocytes and CD19^+^ B cells were determined by a flow cytometer (BD Accuri^TM^ C6). Gating strategy was shown in Supplementary Figure S2.

### 2.7. T Cell Cytotoxic Activity Assay

The P815 cell line at 1 × 10^7^ cells/mL was labeled with 2 µM final concentration of CFSE. The CFSE labeled P815 (4 × 10^4^ cells) were pre-incubated with anti-CD3 mAb or kept in culture medium at 37 °C for 30 min. The CFSE labeled P815 cells (target cells) were co-cultured with PBMCs (effector cells) at effector cells:target cells (E:T) ratios of 2.5:1, 5:1, 10:1, or without effector cells. The CB1 agonist, CB2 agonist, or DMSO were added. The co-cultured cells (at 100 μL final volume) were incubated in a 5% CO_2_ incubator for 24 h. Cells were harvested, washed, and suspended in 7-AAD solution. The percentage of dead target cells (CFSE^+^ 7-AAD^+^) were determined by a flow cytometer (BD Accuri^TM^ C6). Gating strategy and representative flow cytometric data were exhibited in Supplementary Figure S3.

For determination of T cell degranulation, PBMCs were co-cultured with anti-CD3 mAb, pre-incubated P815 cells, or P815 cells at E:T ratios of 10:1. The co-cultured cells (at 100 μL final volume) were incubated with CB1 agonist, CB2 agonist, or DMSO in 10% FBS-RPMI 1640 containing 1 µM monensin and PE-anti-LAMP-1 mAb or PE-isotype-match control mAb. Cells were cultured in a 5% CO_2_ incubator for 24 h. The cells were harvested, washed, fixed and permeabilized. The intracellular staining was performed by adding FITC-anti-CD4 mAb, PEcy7-anti-CD3 mAb, and APC-anti-CD8 mAb. The percentage of LAMP-1 positive cells in CD8^+^ T cells and CD4^+^ T cells was measured by a flow cytometer (BD Accuri^TM^ C6). Gating strategy was shown in Supplementary Figure S4.

For analysis of IFN-γ producing CD8^+^ T cells, the anti-CD3 mAb pre-incubated P815 cells or P815 cells were co-cultured with PBMCs at E:T ratios of 10:1. The cells (at 100 μL final volume) were cultured in the presence of CB1 agonist, CB2 agonist, or DMSO in 10% FBS-RPMI 1640. The cells were kept in a 5% CO_2_ incubator for 24 h. During incubation at 19 h, protein transport inhibitors (1 μg/mL brefeldin A and 1 μM monensin) were added. After 24 h incubation, cells were intracellularly stained with PEcy7-anti-CD3 mAb, APC-anti-CD8 mAb, and PE-anti-IFN-γ mAb or PE-isotype-match control mAb. The stained cells were measured by a flow cytometer (BD Accuri^TM^ C6). Gating strategy for analysis of IFN-γ positive cells was demonstrated in Supplementary Figure S4.

### 2.8. NK Cell Cytotoxic Activity Assay

CFSE labeled K562 cells (target cells) at 5 × 10^4^ cells were co-cultured with PBMCs (effector cells) at E:T ratios of 20:1, 40:1, or without effector cells. The CB1 agonist, CB2 agonist, or relevant concentrations of DMSO were added into the cell culture (at 150 μL final volume) and incubated in a 5% CO_2_ incubator for 4 h. Cells were harvested, washed, and 7-AAD solution was added. The percentage of dead target cells (CFSE^+^ 7-AAD^+^) were measured by a flow cytometer (BD Accuri^TM^ C6).

### 2.9. Intracellular Cytokines Assay

PBMCs were cultured at 1 × 10^6^ cells in 300 µL total volume with immobilized anti-CD3 mAb clone OKT3 (25 ng/mL) and soluble anti-CD28 mAb (25 ng/mL) or kept unstimulated. The CB1 agonist, CB2 agonist, or relevant concentration of DMSO was added and incubated in a 5% CO_2_ incubator for 6 h. During incubation at 1 h, 1 μg/mL brefeldin A and 1 μM monensin were added. Cells were harvested, fixed, permeabilized, and stained with PEcy7-anti-CD3 mAb, and each PE-anti-human cytokine antibody (anti-IFN-γ, -IL-2, or -TNF-α mAb) or PE-isotype-matched control mAb. The intracellular cytokines were determined by a flow cytometer (FACSCelesta). Gating strategy and representative flow cytometric data were exhibited in Supplementary Figure S5.

### 2.10. Statistics

All statistical analyses were performed using Prism 9.2.0 (GraphPad Software, San Diego, CA, USA). Data were expressed as mean ± SD. The unpaired *t*-test or two-way ANOVA were used as indicated in the figure legends. *p* < 0.05 was considered significant.

## 3. Results

### 3.1. CB1 Agonist ACEA and CB2 Agonist GW833972A Impair T Cell Proliferation

**Figure 1:**
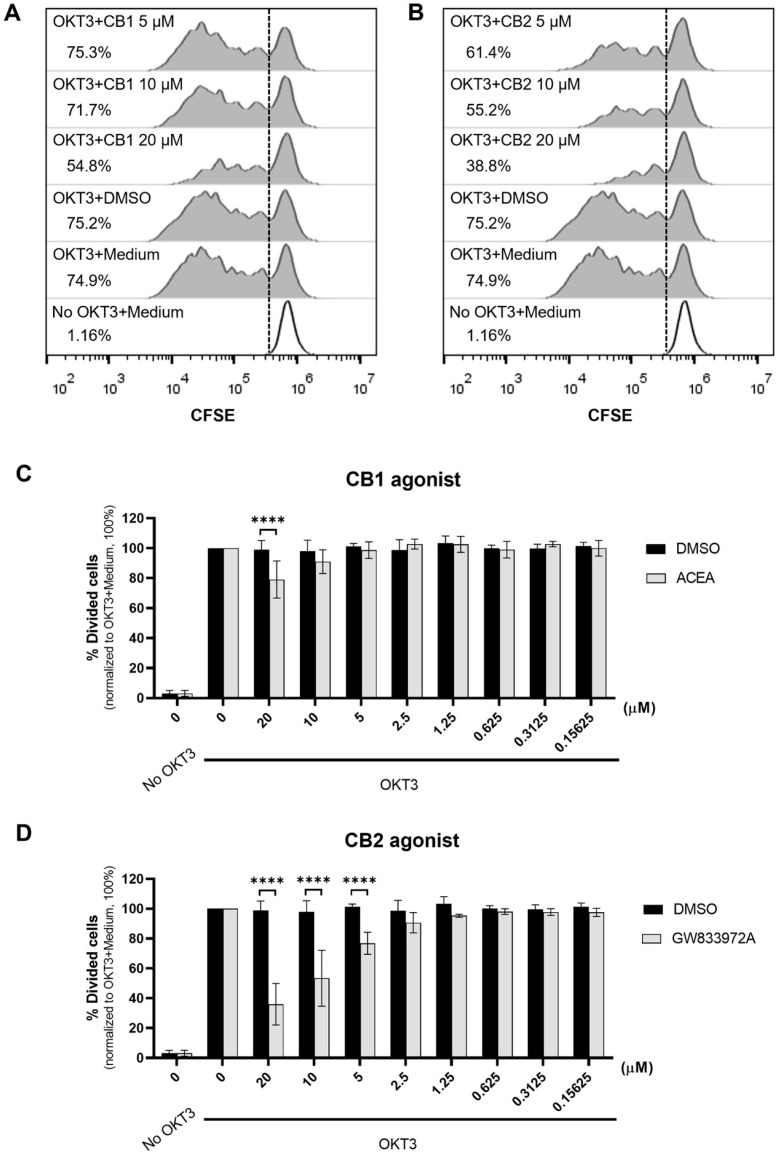
CB1 and CB2 agonists inhibit T cell proliferation. CFSE labeled PBMCs were activated with anti-CD3 mAb (OKT3) or kept unstimulated (No OKT3) in the presence of various concentrations of CB1 (ACEA) or CB2 (GW833972A) agonists or DMSO control (DMSO) for 5 days. The representative flow cytometric data of the CB1 agonist (**A**) and CB2 agonist (**B**) treatment and the relevant DMSO concentration for CB1 or CB2 agonists at 20 µM are expressed in histograms showing the percentage of divided cells in each condition, which was determined using a CFSE proliferation assay. The percentage of divided cells in the presence of the indicated concentrations of CB1 agonist (**C**) and CB2 agonists (**D**) compared with that in the DMSO control is shown in bar graphs as mean ± SD (n = 8 for 0, 20, 10, and 5 µM and n = 4 for other concentrations). Each individual datum was normalized relative to its medium control (OKT3 + Medium) as 100%. We used two-way ANOVA followed by Sidak’s multiple comparisons test for comparison; **** *p* < 0.0001.

We employed the CB1 selective agonist, ACEA [27], and CB2 selective agonist, GW833972A [28], to determine the effect of cannabinoid receptor activation on T cell proliferation. As shown in Figure 1, 20 µM ACEA significantly reduced T cell proliferation compared with the DMSO control, whereas GW833972A impaired T cell proliferation at 5, 10 and 20 µM.

**Figure 2:**
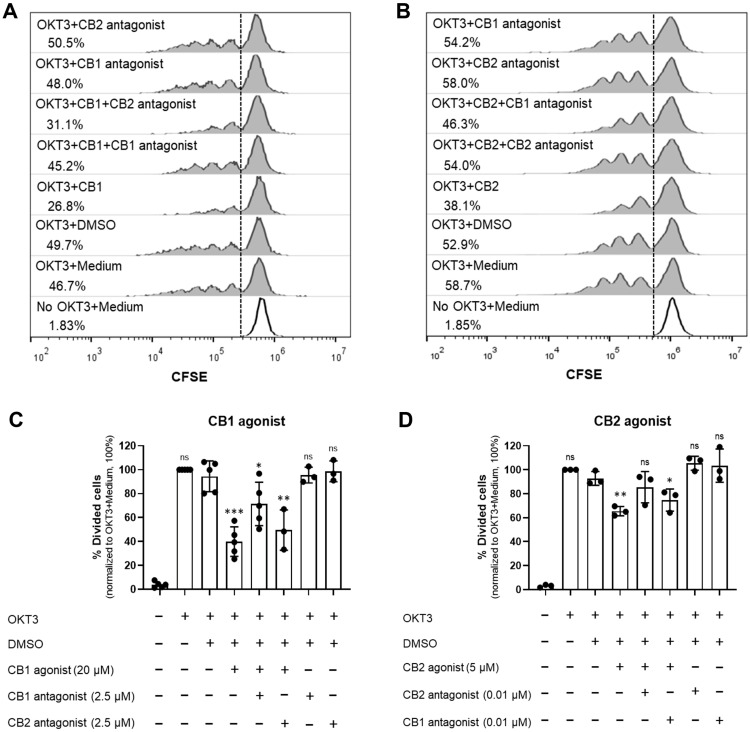
CB1 and CB2 agonists suppress T cell activation via their specific receptors. PBMCs were activated with anti-CD3 mAb (OKT3) or kept unstimulated (No OKT3) in the presence (+) or absence (−) of CB1 agonist (ACEA), CB2 agonist (GW833972A) or their antagonists (CB1 antagonist or CB2 antagonist) at the indicated concentrations or DMSO control (DMSO) at the relevant concentration for agonist plus antagonist. Histograms showing the percentage of divided cells in each condition provide the representative flow cytometric data of CB1 agonist (**A**) and CB2 agonist (**B**) treatment with their antagonists. The bar graphs show the percentage of divided cells under the indicated conditions of CB1 agonist treatment (n = 3 or n = 5) (**C**) and CB2 agonist treatment (n = 3) (**D**) as mean ± SD. A black dot represents individual datum. Each individual datum was normalized relative to its medium control (OKT3 + Medium) as 100%. We used unpaired *t*-test for comparison between DMSO control and each condition. * *p* < 0.05; ** *p* < 0.01; *** *p* < 0.001; ns = not statistically significant.

To confirm whether T cell proliferation inhibition occurred due to the reaction of the agonists and their specific receptors, the we used CB1 antagonist (SR141716A) [27,29] and CB2 antagonist (SR144528) [28,29] to rescue the inhibitory effect of the cannabinoid receptor agonists. Upon T cell activation, SR141716A rescued the inhibitory effect of the CB1 agonist but not the CB2 agonist (Figure 2A,C). Likewise, SR144528 restored the inhibitory effect of the CB2 agonist but not the CB1 agonist (Figure 2B,D). These results indicated that the impaired T cell proliferation was result of the activation of CB1 and CB2 by the tested agonists.

We also investigated the toxicity of the agonists used on T cells. ACEA at 20 µM and GW833972A at 5 µM, which suppressed T cell proliferation, did not show cellular toxicity (Supplementary Figure S6).

### 3.2. CB1 Agonist ACEA and CB2 Agonist GW833972A Differently Altered Activation-Associated Molecules Expressed on T Cells, B Cells, and Monocytes

**Figure 3:**
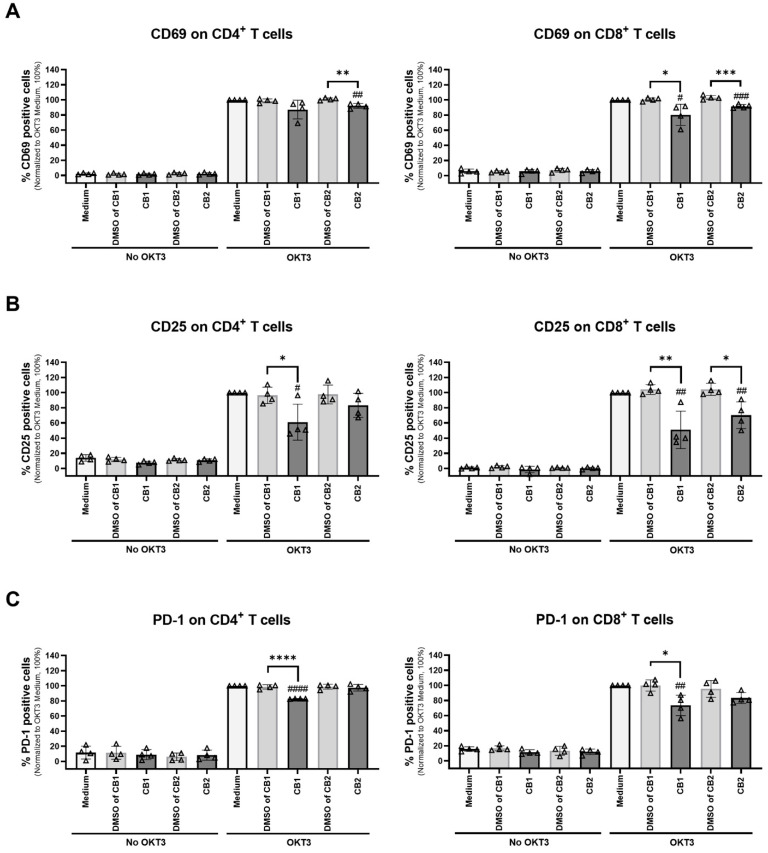
Different effects of CB1 and CB2 agonists on T cell activation molecules. PBMCs (n = 4) were activated with or without anti-CD3 mAb. We analyzed T cell activation markers in the presence of 20 µM CB1 agonist ACEA or 5 µM CB2 agonist GW833972A and compared the results with those of the relevant concentrations of DMSO or medium control. The percentage of CD69- (**A**) and CD25- (**B**) expressing CD4^+^ T cells and CD8^+^ T cells was detected on Day 1. The percentage of PD-1- (**C**) expressing CD4^+^ T cells and CD8^+^ T cells was detected on Day 3. The data were analyzed using flow cytometry as the percentage of positive cells (% specific marker mAb staining–% isotype-matched control mAb staining). The bar graphs report the percentage of positive cells in the indicated conditions as mean ± SD. A triangle represents individual datum. Each individual datum was normalized relative to its medium control (OKT3 + Medium) as 100%. We used an unpaired *t*-test for comparison of the DMSO control and the tested conditions. * *p* < 0.05; ** *p* < 0.01; *** *p* < 0.001; **** *p* < 0.0001. Unpaired *t*-test was used for comparison between the medium control and tested conditions. *^#^ p* < 0.05; *^##^ p* < 0.01; *^###^ p* < 0.001; *^####^ p* < 0.0001.

We determined the effect of CB1 and CB2 agonists on activation molecules, CD69, CD25, and PD-1 expressed on CD4^+^ and CD8^+^ T cells. As shown in Figure 3, upon anti-CD3 mAb activation, ACEA significantly decreased the number of CD25- and PD-1-expressing CD4^+^ T cells, but not for CD69 expression. This agonist decreased the number of CD69-, CD25-, and PD-1-expressing CD8^+^ T cells. For the CB2 agonist, GW833972A significantly diminished the number of CD69-expressing CD4^+^ T cells. GW833972A reduced the number of CD69- and CD25-expressing CD8^+^ T cells. These results suggested that the ACEA and GW833972A altered the expression of T cell activation molecules differently. Without anti-CD3 mAb activation, the CB1 or CB2 agonist alone did not affect activation markers (Figure 3 in No OKT3 panels).

**Figure 4:**
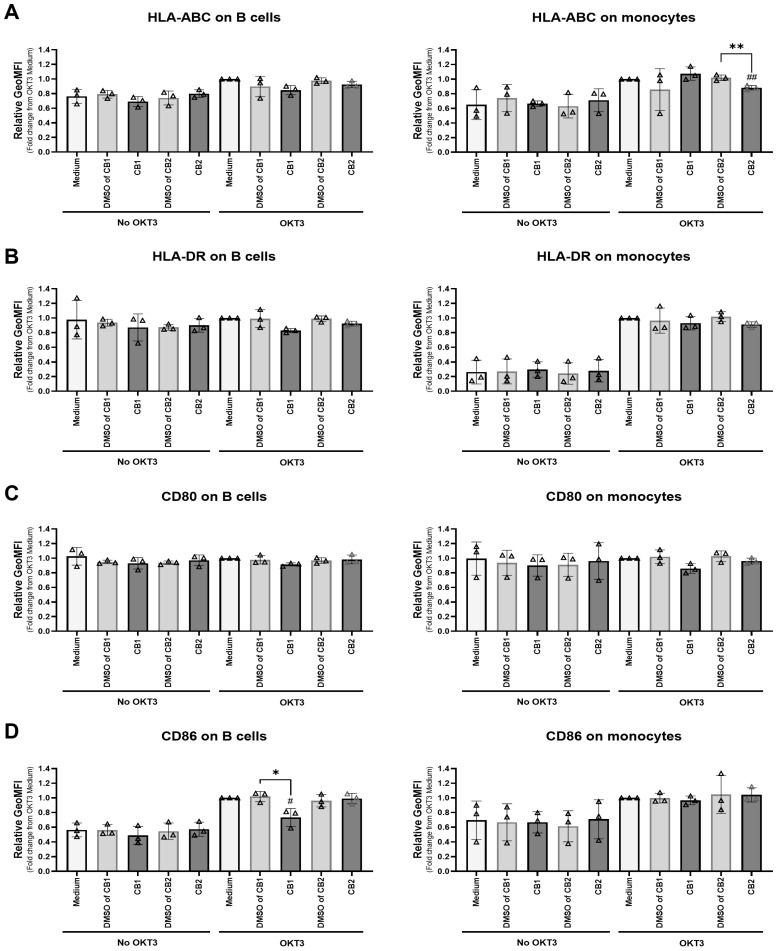
Different effects of CB1 and CB2 agonists on co-stimulatory molecules expressed on B cells and monocytes. PBMCs were stimulated with or without anti-CD3 mAb in the presence of 20 µM CB1 agonist ACEA or 5 µM CB2 agonists GW833972A and the results compared with those of the relevant concentrations of DMSO or medium control for 18 h. The surface expression levels of co-stimulatory molecules HLA-ABC (n = 3) (**A**), HLA-DR (n = 3) (**B**), CD80 (n = 3) (**C**), and CD86 (n = 3) (**D**) on CD19^+^ B cells and CD14^+^ monocytes were determined by flow cytometry. The relative geometric mean fluorescence intensity (GeoMFI of specific marker mAb staining/GeoMFI of isotype-matched control mAb staining) was normalized to the OKT3 and medium control condition as 1. The bar graphs show the relative GeoMFI (mean ± SD), where a triangle represents an individual datum. We used the unpaired *t*-test for comparing the DMSO control and tested condition results. * *p* < 0.05; ** *p* < 0.01. We used an unpaired *t*-test for comparing the medium control and tested conditions. *^#^ p* < 0.05; *^##^ p* < 0.01.

We investigated the effect of ACEA and GW833972A on co-stimulatory molecules expressed on B cells and monocytes. Upon anti-CD3 mAb activation, the CB1 agonist ACEA significantly reduced the expression level of CD86 on B cells (Figure 4D), whereas the CB2 agonist GW833972A decreased the HLA-ABC expression level on monocytes (Figure 4A).

Taken together, these results indicated that the activation of CB1 and CB2 by the tested agonists altered activation-associated molecules and co-stimulatory molecules expressed on T cells, B cells, and monocytes differently.

### 3.3. CB2 Receptor Agonist GW833972A Inhibits Th1 Cytokines Production

**Figure 5:**
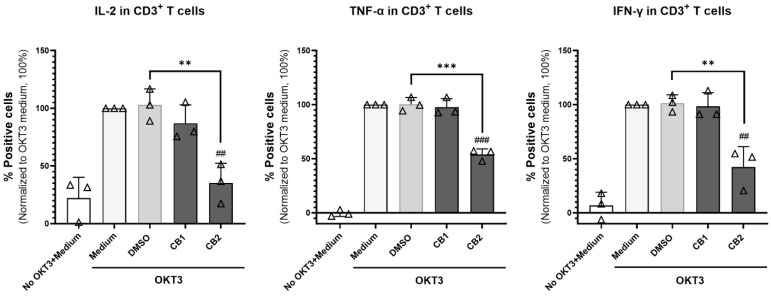
Effect of CB1 and CB2 agonists on cytokine production of T cells. PBMCs (n = 3) were activated with anti-CD3 mAb OKT3 and anti-CD28 mAb in the presence of 20 µM CB1 agonist ACEA or CB2 agonists GW833972A and the results compared with those of the relevant concentration of DMSO or medium control for 6 h. The percentage of cytokine positive cells (% specific marker mAb staining–% isotype-matched control mAb staining) as indicated was determined in the CD3^+^ T cell population by intracellular staining and flow cytometry. The bar graphs report the percentage of cytokine positive cells under the indicated conditions as mean ± SD. A triangle represents an individual datum. Each individual datum was normalized relative to its medium control (OKT3 + Medium) as 100%. We used unpaired *t*-test for comparison of the DMSO control with the tested conditions. ** *p* < 0.01; *** *p* < 0.001. We used unpaired *t*-test to compare the medium control and the tested conditions. *^##^ p* < 0.01; *^###^ p* < 0.001.

We determined intracellular cytokine production in T cells upon stimulation of PBMCs using mAb OKT3 and anti-CD28 mAb. The CB2 agonist GW833972A, but not CB1 agonist ACEA, significantly reduced Th1 cytokine (IL-2, TNF-α, and IFN-γ) production (Figure 5).

### 3.4. CB2 Agonist GW833972A Diminishes Cytotoxic Function of CD8^+^ T Cells but Not NK Cells

**Figure 6:**
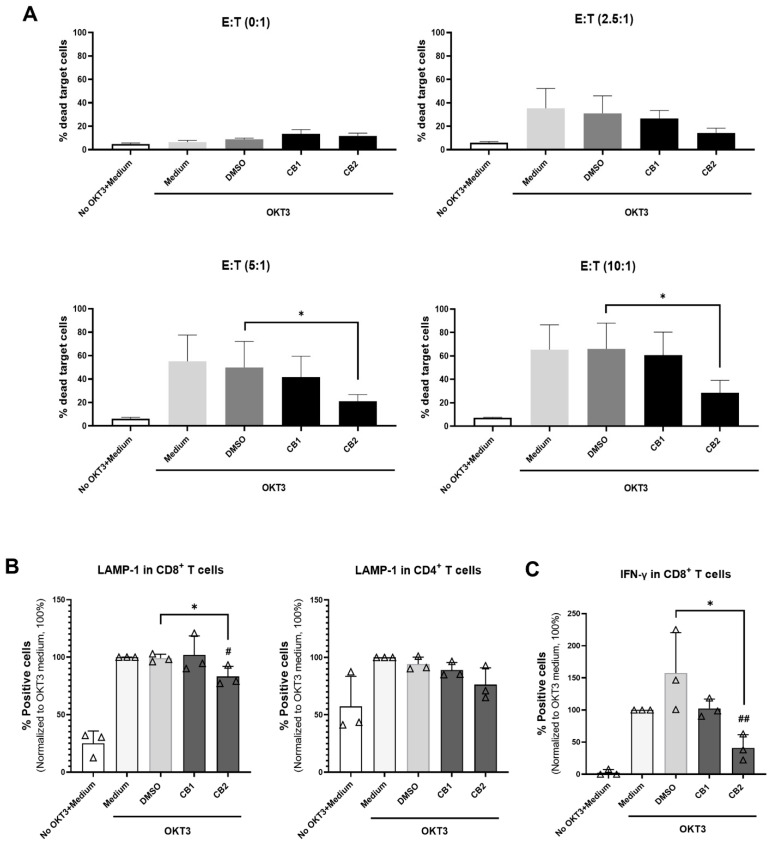
Effect of CB1 and CB2 agonists on cytotoxic function of T cells. We used CFSE-labeled P815 cells as target cells and investigated cytotoxic activity of T cells by co-culture of PBMCs (effector cells) with anti-CD3 mAb OKT3 and P815 target cells at various effector to target (E:T) ratios in the presence of 20 µM CB1 agonist ACEA or CB2 agonists GW833972A, the relevant concentration of DMSO, or medium control for 24 h. (**A**) The bar graphs report the percentage of dead target cells (CFSE^+^7-AAD^+^) as mean ± SD (n = 3). (**B**) The bar graphs exhibit the percentage of LAMP-1 positive in CD8^+^ T cells and CD4^+^ T cells (n = 3) and (**C**) IFN-γ- positive in CD8^+^ T cells (n = 3). The data were analyzed as the percentage of positive cells (% specific marker mAb staining–% isotype-matched control mAb staining). The bar graphs report the percentage of positive cells under the indicated conditions as mean ± SD. A triangle represents an individual datum. Each individual datum was normalized relative to its medium control (OKT3 + Medium) as 100%. All experiments, used unpaired *t*-test to compare the DMSO control with the tested conditions. * *p* < 0.05. We used unpaired *t*-test to compare the medium control and tested conditions. *^#^ p* < 0.05; *^##^ p* < 0.01.

We determined the effect of ACEA and GW833972A on the cytotoxic function of T cells. We used the anti-CD3-primed P815 cell line as target cells for stimulation of T cell-mediated cytotoxic activity [30,31]. CB2 agonist GW833972A, decreased T cell-mediated cytotoxic activity at E:T 5:1 and 10:1 (Figure 6A). At the concentration of GW833972A used, no cytotoxicity to the effector cells was observed (Supplementary Figure S7). However, we did not observe this effect using the CB1 agonist ACEA (Figure 6A). We further determined the degranulation process of CD8^+^ and CD4^+^ T cells upon CB2 activation. As shown in Figure 6B, GW833972A significantly reduced LAMP-1- expressing CD8^+^ T cells but did not affect CD4^+^ T cells. Correspondingly, the number of IFN-γ- producing CD8^+^ T cells were significantly decreased by GW833972A (Figure 6C). These results suggested that the CB2 agonist GW833972A affects the cytotoxic function, degranulation, and IFN-γ production of CD8^+^ T cells.

We also determined the cytotoxic function of NK cells. The tested CB1 and CB2 agonists did not affect NK cell cytotoxicity (Supplementary Figure S8). The results indicated that the attenuation of cytotoxicity by GW833972A occurred on CD8^+^ cytotoxic T cells rather than NK cells.

## 4. Discussion

The endocannabinoid system is involved in immune function [10,13,14,15,32,33,34,35]. Cannabinoid receptors, CB1 and CB2, are expressed on both CD4^+^ and CD8^+^ T cells and are upregulated upon T cell activation by anti-CD3/CD28 mAb [13,36,37]. In this study, we investigated the effects of the CB1 agonist ACEA and CB2 agonist GW833972A on T cell regulation. We found that the activation of CB1 and CB2 by either ACEA- or GW833972A-induced T cell hypofunction in different manners.

ACEA is a high-affinity agonist of the CB1 (K*_i_* values of 1.4–5.29 nM) with a 2000-fold higher selectivity for the CB1 than for the CB2 [27,38]. The effects of ACEA could be inhibited by the CB1 antagonist SR141716A [27,39,40]. GW833972A is a β-arrestin-biased agonist with a 1000-fold higher selectivity for the CB2 than for the CB1 [28,41]. The effects of GW833972A were blocked by the CB2 antagonist SR144528, but not by the CB1 antagonist rimonabant [28]. In this study, we used the CB1 selective agonist ACEA [27] and CB2 selective agonist GW833972A [28] for activation of their specific receptors and determined T cell responses upon activation. We found that the CB1 agonist ACEA at 20 µM reduced T cell proliferation, this effect was not observed at the lower concentrations. This result, however, is different from the previous finding that ACEA did not affect T cell proliferation [13]. Concentration of ACEA used, however, differed between the studies. The previous study used 1 µM; at this concentration, we also found no effect on T cell proliferation. In our study, ACEA at 20 µM significantly reduced T cell proliferation; however, this concentration was not tested in the previous study [13]. Therefore, the effect of ACEA on T cell proliferation in the previous study might be omitted [13]. Moreover, in our study, we used anti-CD3 activated PBMCs while the previous study used purified T cells [13]. In the present study, we demonstrated that ACEA decreased the co-stimulatory molecule CD86 on B cells. Decreasing the co-stimulatory molecule may involve in the reduction of T cell proliferation. As purified T cells were used in the previous study [13], which lack B cells, the effect of ACEA on T cell proliferation may not be observed. We suggested that activation of CB1 by ACEA is involved in the regulation of the T cell response. To the best of our knowledge, this is the first time this finding has been reported.

To investigate the involvement of CB2 on T cell regulation in this study, GW833972A was used for CB2 activation. GW833972A had been used to determine the role of CB2 in airway sensory nerve function for the treatment of chronic cough [28]. However, the effects of GW833972A on T cell responses have not been tested. Our results demonstrated that, as same as the CB1 agonist ACEA, GW833972A impaired T cell proliferation.

We nevertheless raised the question whether the observed effect of ACEA and GW833972A on T cell proliferation was actually due to the interaction of cannabinoid receptors with their specific agonists. To address this question, we used CB1 antagonist SR141716A and CB2 antagonist SR144528 [29] to neutralize the effect of the tested agonists. The inhibitory effect of CB1 and CB2 agonists on T cell proliferation was restored by their specific antagonists. According to the different K*_i_* of the CB1 antagonist SR141716A (1.98 nM) and CB2 antagonist SR144528 (0.6 nM), the ratio of agonist to antagonist are at 1:8 for CB1 and 1:500 for CB2. These results confirmed that the inhibitory effects of ACEA and GW833972A occurred through the binding and activation of CB1 and CB2, respectively. We, therefore, suggest that both cannabinoid receptors play a role in T cell responses. The activation of CB1 and CB2 by ACEA and GW833972A, respectively, suppressed T cell proliferation upon stimulation.

We elucidated the biological mechanisms of ACEA and GW833972A involving the regulation of T cell responses. We found that the activation of CB1 and CB2 by ACEA and GW833972A attenuated T cell proliferation through different mechanisms. Upon T cell activation, the T cell activation molecules, including CD69, CD25, and PD-1, were upregulated on the T cell surface [42,43,44,45,46]. The CB1 selective agonist ACEA decreased the number of CD4^+^ T cells expressing CD25 and PD-1, while it decreased the number of CD8^+^ T cells expressing CD69, CD25, and PD-1. The CB2 selective agonist GW833972A significantly diminished the number of CD4^+^ T cells expressing CD69 but had no effect on CD25 and PD-1 expression, as well as the number of CD8^+^ T cells expressing CD69 and CD25. These results indicated that the agonists ACEA and GW833972A affect CD4^+^ and CD8^+^ T cells differently. In addition, we determined the effect of ACEA and GW833972A on B cells and monocytes. Monocytes and B cells are antigen presenting cells and express co-stimulatory molecules including HLA-ABC (MHC class I), HLA-DR (MHC class II), CD80, and CD86, which contribute to T cell activation [47,48]. Downregulation of these co-stimulatory molecules was demonstrated to attenuate T cell functions [49,50]. We observed that the CB1 agonist ACEA significantly reduced the expression levels of CD86 on B cells, whereas the CB2 agonist GW833972A decreased HLA-ABC expression levels on monocytes, which might have resulted in reduction of T cell proliferation. These results indicated that ACEA and GW833972A affect different APCs for attenuating T cell responses. However, in this study, the mechanisms regulated by CB1 or CB2 activation in each cell type have not been investigated. It is also likely that the CB1 and CB2 expression levels in each cell type were differentially changed after OKT3 stimulation. There is a report that demonstrated that CB1 was upregulated on macrophages upon PMA stimulation leading to an increasing CB1:CB2 ratio. By changing the CB1:CB2 ratio, the opposing outcomes mediated by CB1 and CB2 were found in which CB2 is a negative regulator of CB1-stimulated ROS production [51].

Furthermore, we determined the effect of ACEA and GW833972A on Th1 cytokine (IL-2, TNF-α, and IFN-γ) production. Th1 cytokine are cytokines that are required for boosting immune cells to eliminate invading pathogens and tumor cells [52,53]. We found that only GW833972A decreased the number of T cell producing Th1 cytokines. This CB2 agonist, however, did not affect the production of IL-17 by T cells and IL-10, TNF-α, and IL-6 by monocytes. The results indicated that the agonists used did not produce cytotoxicity. This result is related to the effects of the CB2 agonist, which suppressed T cell proliferation and inhibited the production of IFN-γ and TNF-α through a CB2-dependent pathway [11,54,55]. CD8^+^ cytotoxic T cells and NK cells play an important role in eliminating virus-infected cells and tumor cells. Additionally, CD4^+^ cytotoxic T cells have been demonstrated to play an important role in antitumor immunity [56,57,58]. These cytotoxic cells can recognize tumor cells and induce tumor cell death by releasing cytolytic granules and cytokines, particularly IFN-γ [56,57,58]. The CB1 and CB2 have been reported to be expressed on T cells and NK cells [5,36]. In this study, we investigated the cytotoxic function of T cells and NK cells upon cannabinoid receptor activation by ACEA and GW833972A. The CB2 selective agonist GW833972A, but not CB1 agonist ACEA, suppressed the cytotoxic activity, IFN-γ production, and degranulation process of CD8^+^ cytotoxic T cells, whereas the cytotoxic functions of CD4^+^ cytotoxic T cells and NK cells were not affected by the tested CB1 or CB2 agonist. CB2 is expressed in various peripheral tissue; however, its expression is higher in the immune system [5,17,59]. Activation of CB2 modulates various intracellular signal transduction pathways, including inhibition of adenyl cyclase activity to produce cAMP and an increase in the phosphorylation of MAPK [60,61,62]. The CB2 was demonstrated to be involved in the suppression of T cell functions, including suppression of T cell activation [63,64,65,66], reduction of cytokine production [11,55], and migration [67]. In agreement with our results, the CB2 selective agonist GW833972A suppressed the cytotoxic activity of CD8^+^ T cells.

Taken together, we demonstrated that the CB1 agonist ACEA and CB2 agonist GW833972A contribute to the immune-regulation process but in different biological mechanisms. These cannabinoid receptor agonists impaired T cell functions. We emphasize that the activation of cannabinoid receptors by the agonists used in this study may lead to a treatment for autoimmune and inflammatory diseases.

## Data Availability

Most data generated or analyzed during this study are included in this published article and its Supplementary Materials.

## Supplementary Materials

The following supporting information can be downloaded at: https://www.mdpi.com/article/10.3390/cells12060848/s1. Figure S1: Gating strategy for analysis of T cell activation markers; Figure S2: Gating strategy for analysis of co-stimulatory molecules expressed on monocytes and B cells; Figure S3: Gating strategy and a representative flow cytometric data of cytotoxic function of CD8^+^ T cells; Figure S4: Gating strategy for analysis of LAMP-1 and IFN-γ positive cells; Figure S5: Gating strategy and a representative flow cytometric data of T helper cytokines; Figure S6: Investigation of toxicity of CB1 and CB2 agonists on T cell proliferation; Figure S7: A representative flow cytometric data of cytotoxic function of CD8^+^ T cells; Figure S8: Effect of CB1 and CB2 agonists on cytotoxic function of NK cells.
