# Supplementary material for: Cannabinoid Receptor 1 Agonist ACEA and Cannabinoid Receptor 2 Agonist GW833972A Attenuates Cell-Mediated Immunity by Different Biological Mechanisms
Source: Cells. 2023 Mar 9;12(6):848. doi: 10.3390/cells12060848 (PMC10047765; doi:10.3390/cells12060848)
Supplement: Supplementary file 1 [file cells-12-00848-s001.zip › cells-2217547-supplementary.pdf]

Supplementary material

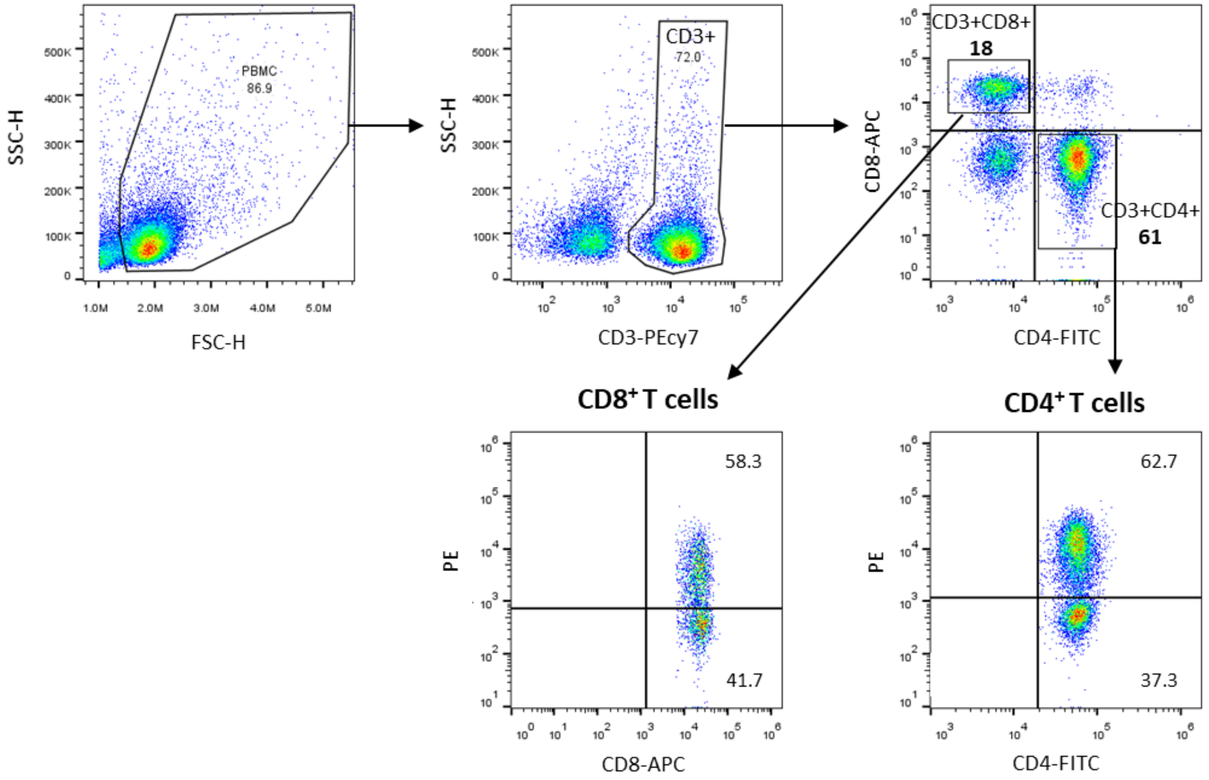

Figure S1. Gating strategy for analysis of T cell activation markers. PBMCs were gated from FSC-H and SSC-H to analysis of $CD3^+$ T cells. The $CD8^+$ and $CD4^+$ T cells were further gated from $CD3^+$ T cells to analysis of T cell activation marker positive cells (PE). The number expressed in dot plot indicates the percentage of cells.

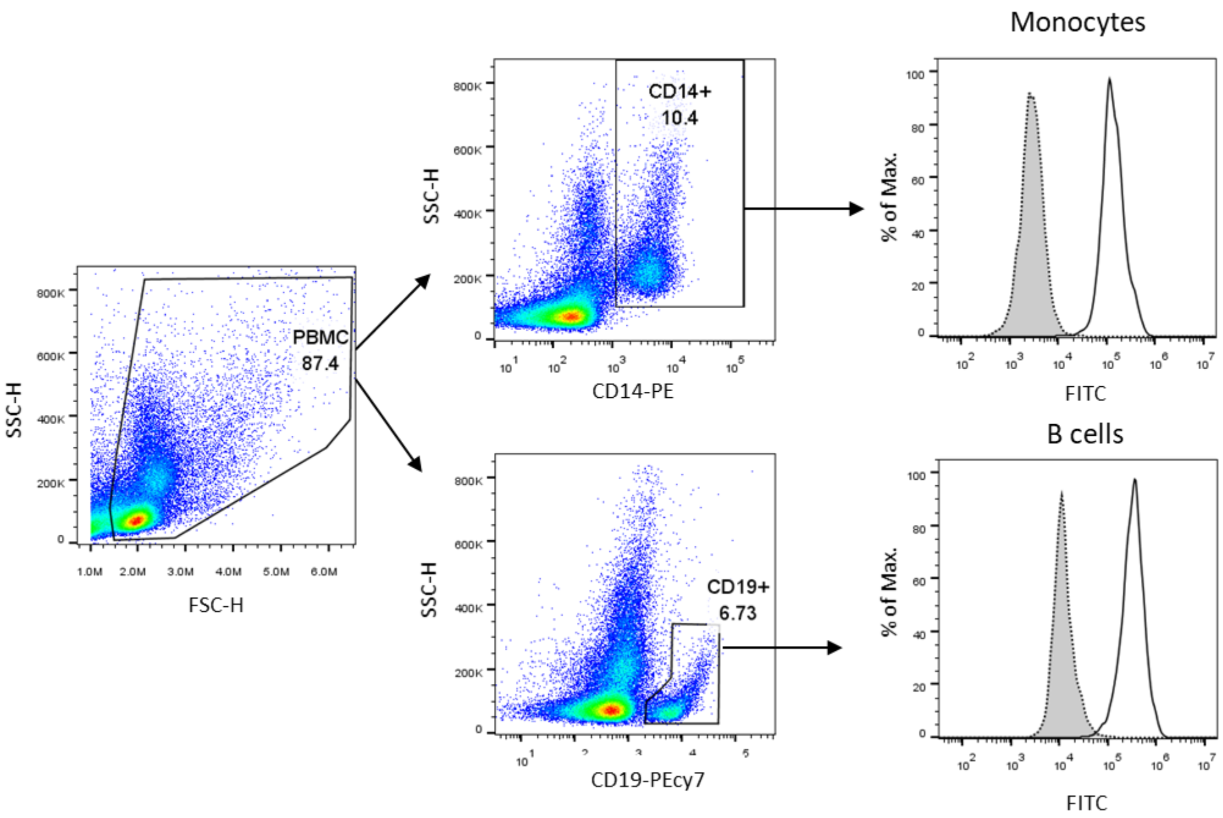

Figure S2. Gating strategy for analysis of co-stimulatory molecules expressed on monocytes and B cells. PBMCs were gated from FSC-H and SSC-H to analysis of $CD14^+$ monocytes and $CD19^+$ B cells. The surface expression levels (FITC) of specific marker mAb staining (White peak) and isotype-matched control mAb staining (Gray peak) on $CD14^+$ monocytes and $CD19^+$ B cells were exhibited in over layered histograms.

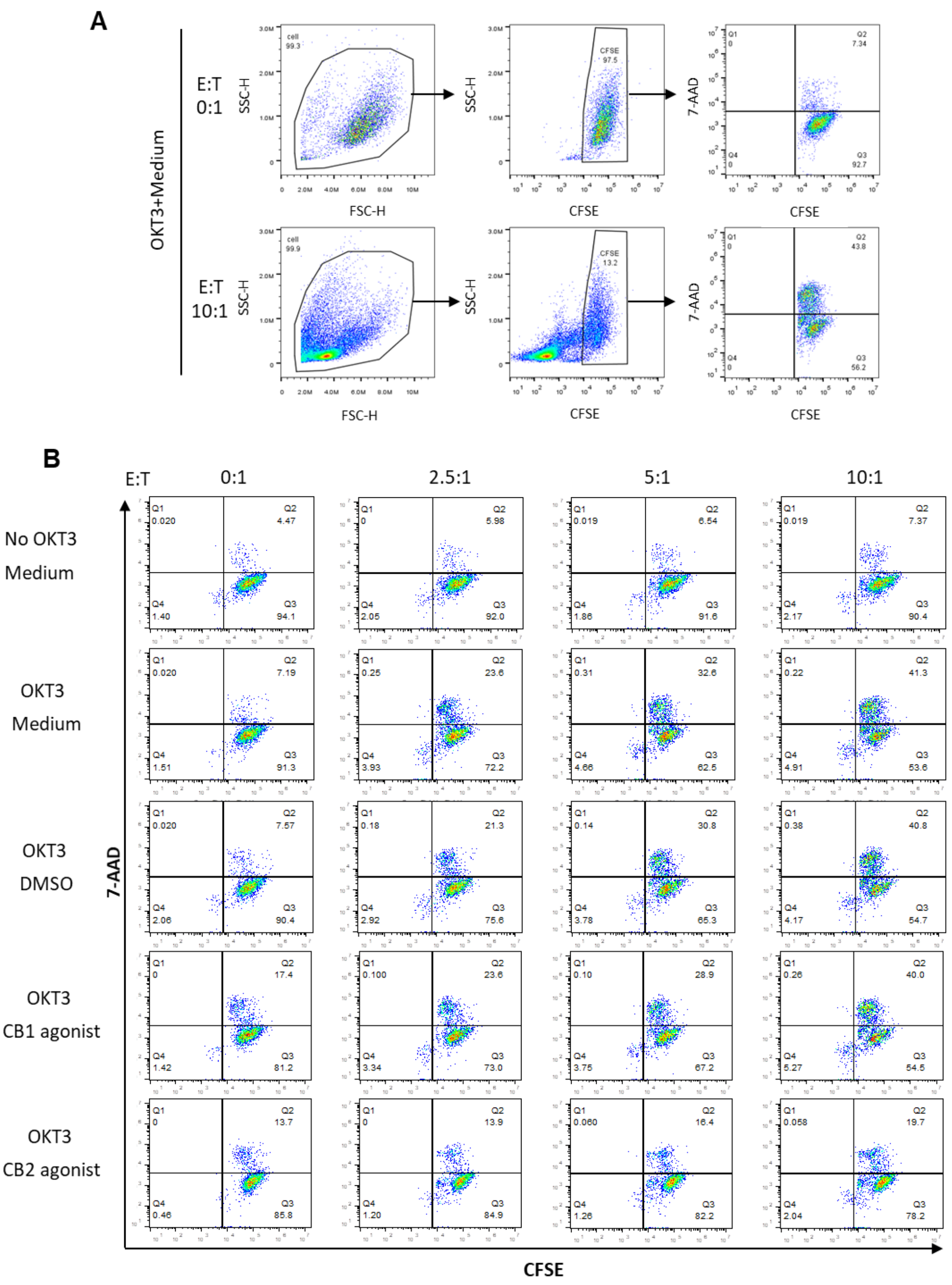

Figure S3. Gating strategy and a representative flow cytometric data of cytotoxic function of CD8$^+$ T cells. (A) The dot plot showing gating strategy at E:T ration of 0:1 and 10:1. All cells were gated from FSC-H and SSC-H to analysis of CFSE labeled target cells. The CFSE labeled target cells were further gated for determining the percentage of dead target cells (CFSE$^+$7-AAD$^+$). (B) A representative flow cytometric data from three individuals in the indicated conditions is exhibited.

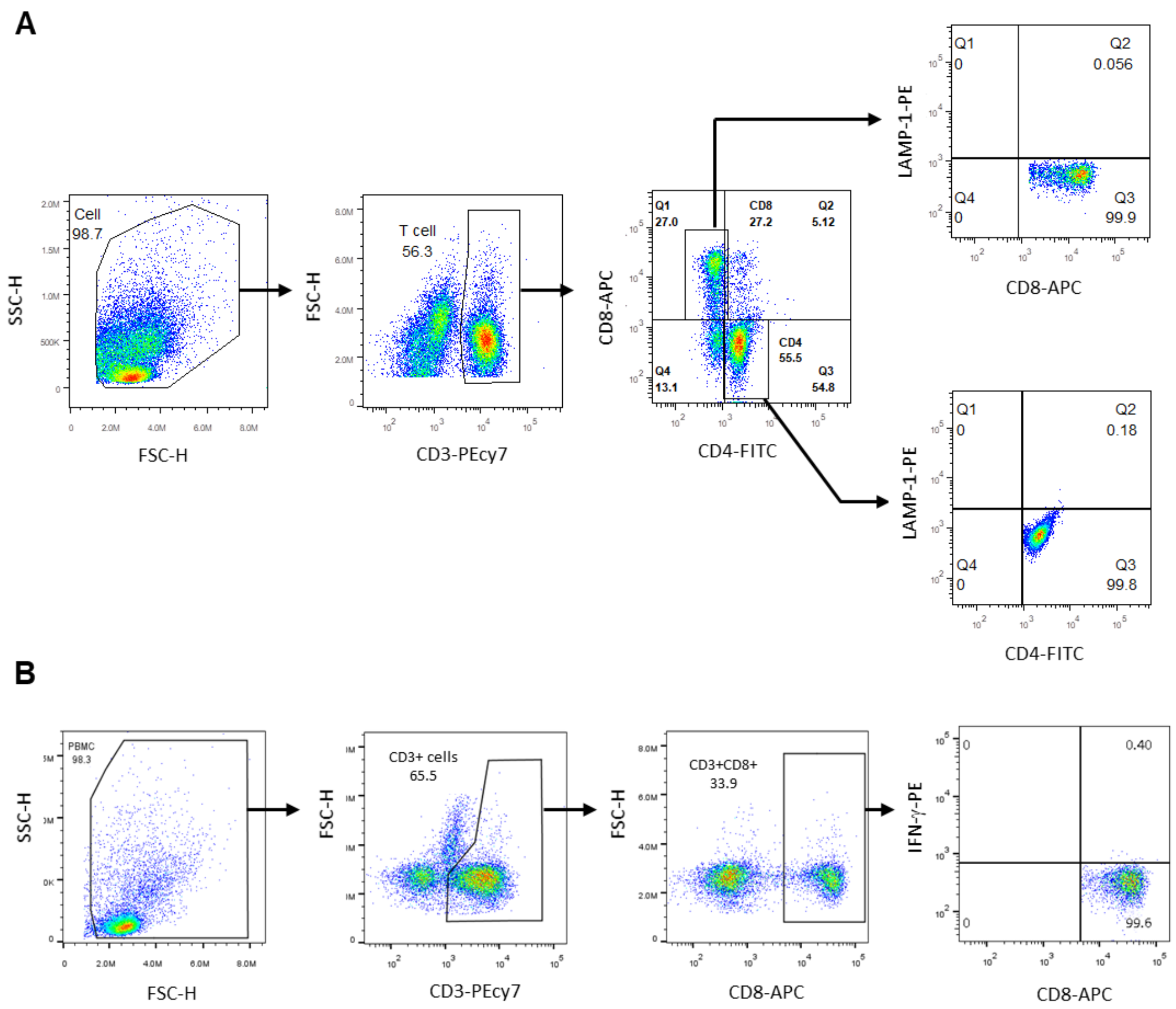

Figure S4. Gating strategy for analysis of LAMP-1 and IFN-γ positive cells. (A) PBMCs were gated from FSC-H and SSC-H to analysis of $CD3^+$ T cells. The $CD8^+$ and $CD4^+$ T cells were further gated from $CD3^+$ T cells to analysis of the percentage of positive cells (LAMP-1-PE). The percentage of positive cells was set up from negative isotype-matched control mAb staining showing in last column. (B) PBMCs were gated from FSC-H and SSC-H to analysis of $CD3^+$ T cells. The $CD8^+$ T cells were further gated from $CD3^+$ T cells to analysis of the percentage of positive cells (IFN-γ-PE). The percentage of positive cells was set up from negative isotype-matched control mAb staining showing in last column.

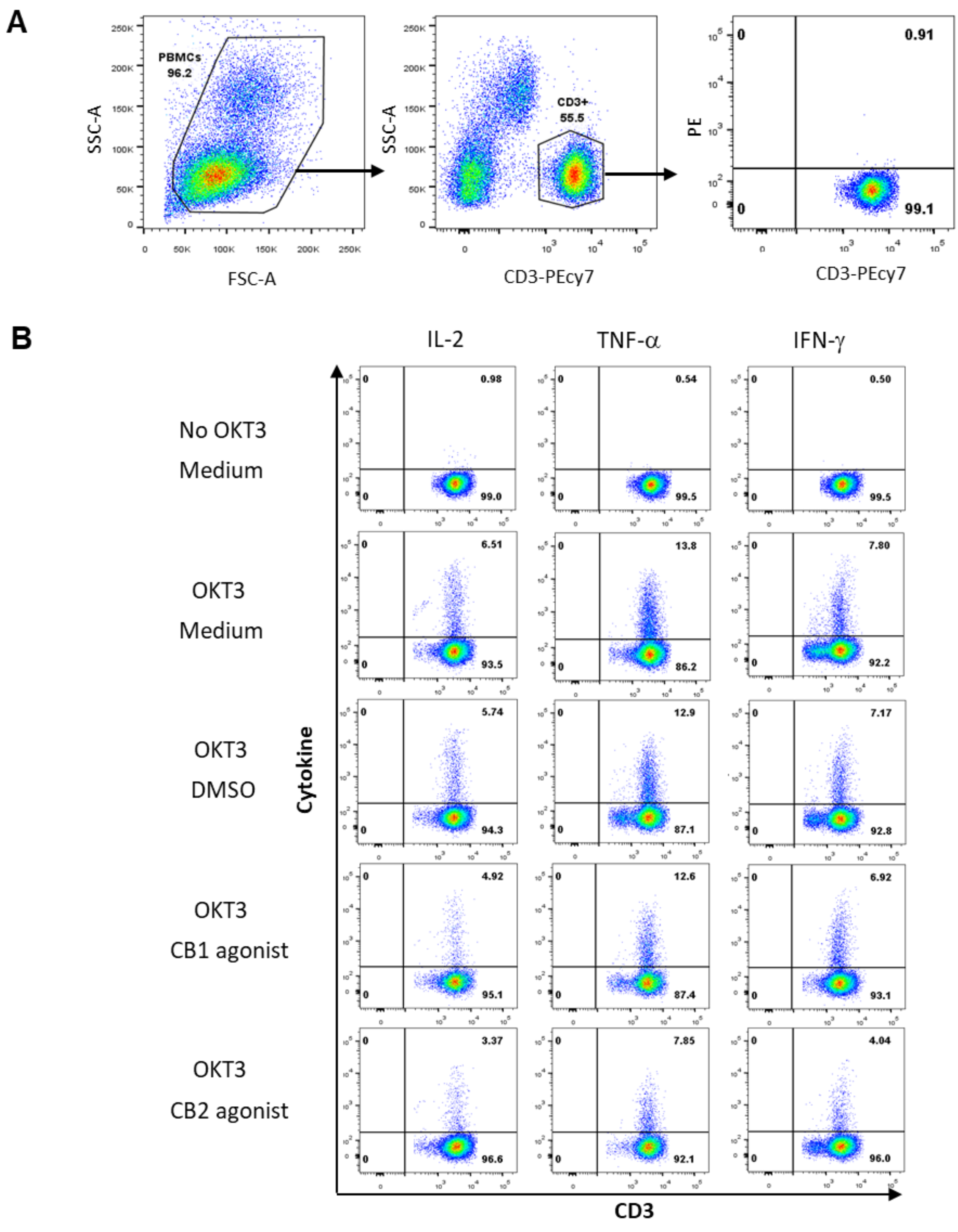

Figure S5. Gating strategy and a representative flow cytometric data of T helper cytokines. (A) PBMCs were gated from FSC-A and SSC-A to analysis of $CD3^+$ T cells. The percentage of cytokine positive T cells (PE) was set up from negative isotype-matched control mAb staining showing in last column. (B) A representative flow cytometric data from three individuals in the indicated conditions is exhibited.

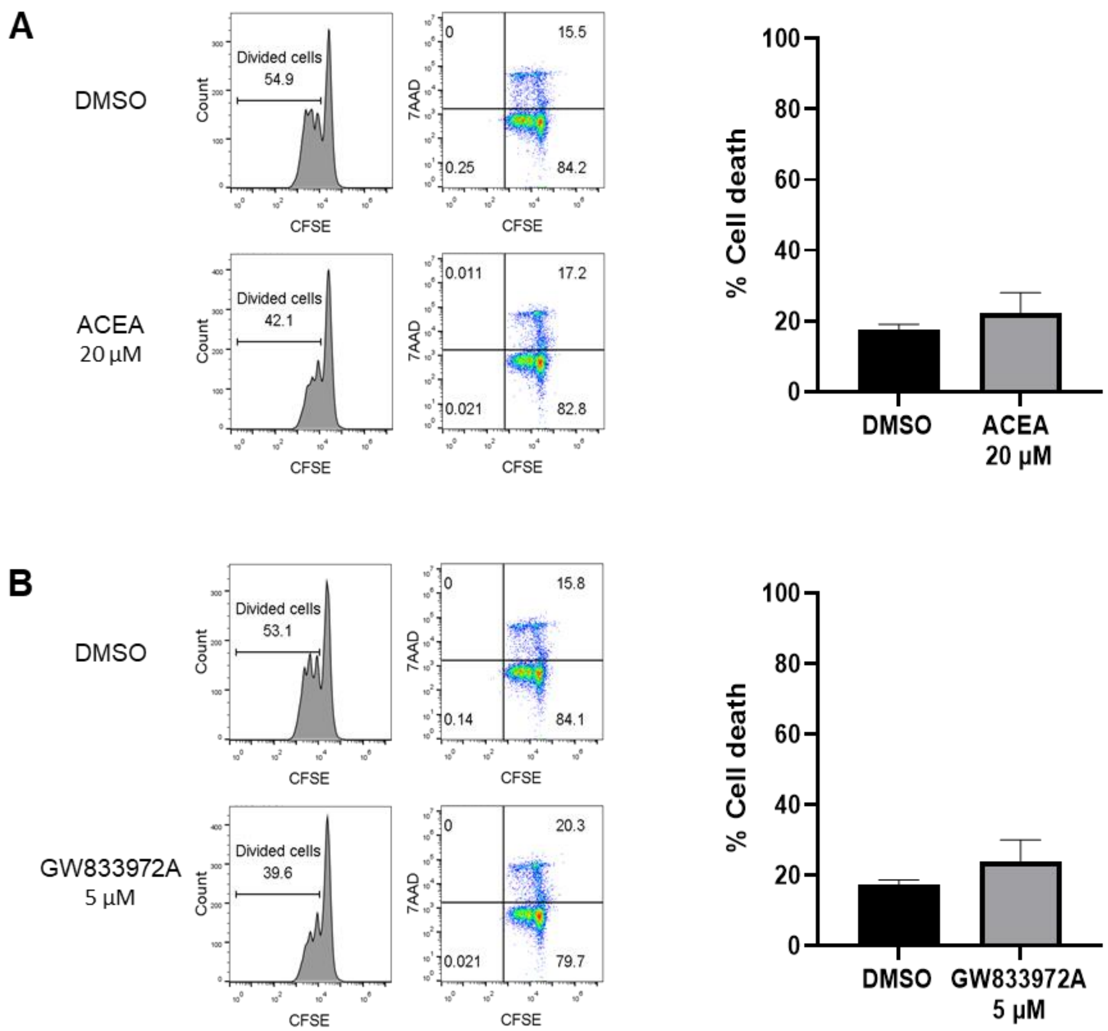

Figure S6. Investigation of toxicity of CB1 and CB2 agonists on T cell proliferation. CFSE labeled PBMCs were activated with anti-CD3 mAb OKT3 and ACEA 20 µM or GW833972A 5 µM or relevant concentration of DMSO for 5 days. Toxicity was determined by 7-AAD staining. The toxicity results of CB1 agonist (A) or CB2 agonist (B) are shown. Flow cytometric analysis is a representative data from three individuals showing the percentage of divided cells (Left panel) and the percentage of cell death or 7-AAD positive cells (Right panel). The bar graph expresses as mean ± SD of the percentage of cell death of three individuals. No statistical difference in the induction of cell death by ACEA and GW833972A in comparison to their relevant concentration of DMSO.

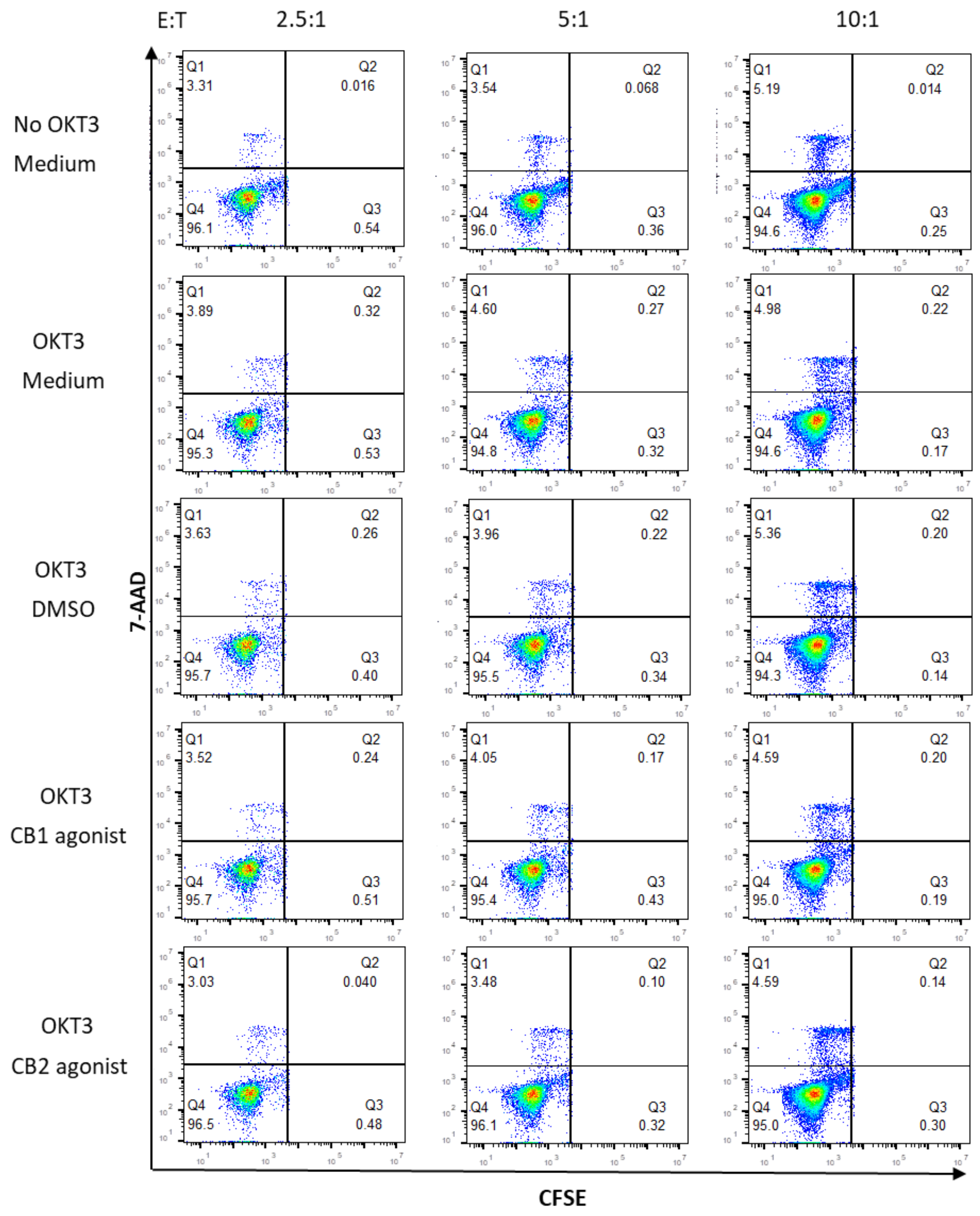

Figure S7. A representative flow cytometric data of cytotoxic function of $CD8^+$ T cells. The CFSE labeled P815 cells (target cells) were co-cultured with PBMCs (effector cells) at effector cells:target cells (E:T) ratios of 2.5:1, 5:1, 10:1. The CB1 agonist, CB2 agonist, or DMSO were added. After incubation, cells were suspended in 7-AAD solution. The percentage of dead effector cells ($CFSE^-$ $7\text{-}AAD^+$) were determined by a flow cytometer. All cells were first gated from FSC-H and SSC-H to analysis of CFSE negative effector cells. The CFSE negative effector cells were further gated for determining the percentage of dead effector cells ($CFSE^-7\text{-}AAD^+$). A representative flow cytometric data from three individuals in the indicated conditions is exhibited. No statistical difference in the induction of cell death by ACEA and GW833972A in comparison to relevant concentration of DMSO and medium control.

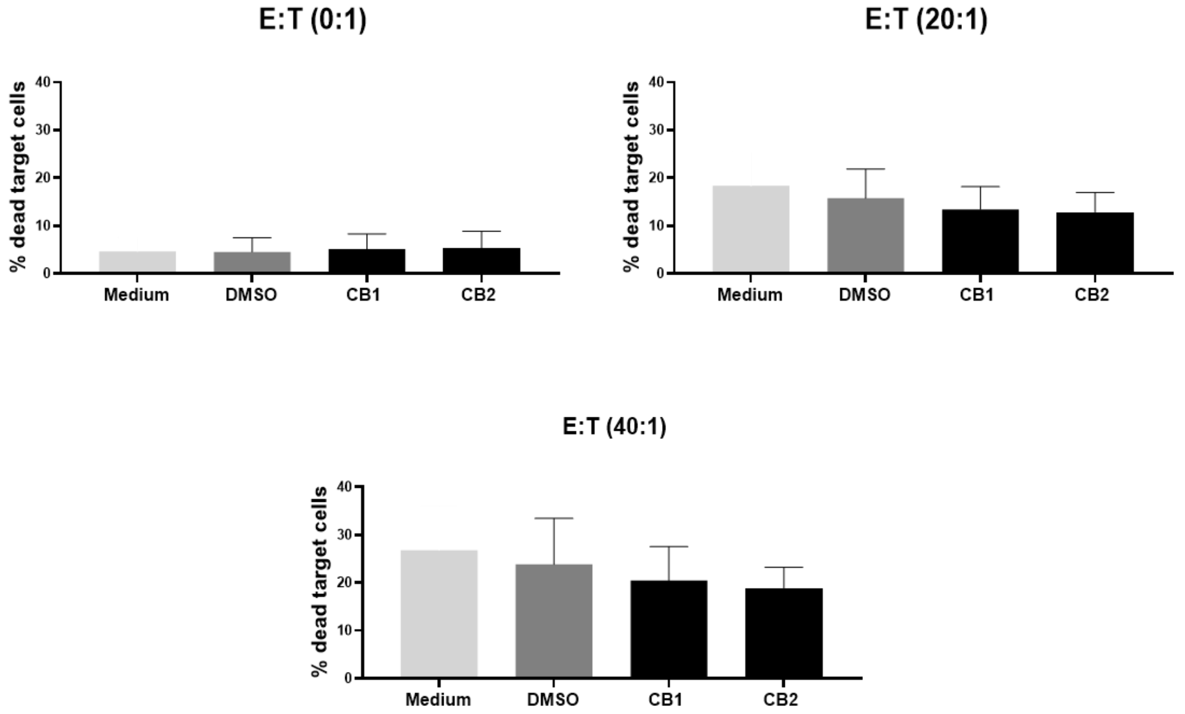

Figure S8. Effect of CB1 and CB2 agonists on cytotoxic function of NK cells. CFSE labeled K562 cells were used as target cells. The cytotoxic activity of NK cells was investigated by co-culture of PBMCs (Effector cells) with K562 target cells at various effector to target (E:T) ratio in the presence of 20 µM CB1 agonist ACEA, CB2 agonist GW833972A, or relevant concentrations of DMSO for 4 hours. The target cell death was determined by 7-AAD staining and the percentage of dead target cells ($CFSE^{+}$7-AAD^{+}) were analyzed by flow cytometry. The bar graphs expressing percentage of dead target cells ($CFSE^{+}$7-AAD^{+}) were mean ± SD (n=3).
